# Standardizing smokeless tobacco packs in India to enhance health warning visibility and harm perceptions

**DOI:** 10.18332/tid/205097

**Published:** 2025-06-30

**Authors:** Hannah E. Barker, Raniyan Zaman, Lauren Czaplicki, Sejal Saraf, Rana J. Singh, Ashish K. Pandey, Joanna E. Cohen

**Affiliations:** 1 Institute for Global Tobacco Control, Department of Health, Behavior and Society, Johns Hopkins Bloomberg School of Public Health, Baltimore, United States; 2 Vital Strategies Tobacco Control Division, New Delhi, India; 3 Vital Strategies Tobacco Control Division, New York, United States

**Keywords:** policy, smokeless tobacco, health warning label, tobacco packaging, India

## Abstract

**INTRODUCTION:**

Approximately 21% of adults in India use smokeless tobacco and over half use khaini, a tobacco-lime mixture. Khaini is available in a variety of pack shapes and sizes, which can affect health warning label (HWL) size and placement. This study explored consumer perceptions of existing khaini packs and two proposed standard shape/size khaini packs across dimensions of HWL noticeability and harm perceptions.

**METHODS:**

In March 2023, we conducted 24 focus groups (FGs) across India. Groups were equally numbered by residence, gender, and current khaini use. FGs were led by trained facilitators. Each FG discussed six existing khaini packs and two standard packs (paper sachet; tin cylinder). Data were collected in local languages, translated into English, and thematically analyzed.

**RESULTS:**

All FGs discussed the limited visibility of HWLs on existing packs, noting that HWLs were often small, blurry, or printed too faintly to notice. Most (defined as ≥80%) FGs discussed how the HWLs on both standard packs were large and easy to see. FGs discussed how the standard packs appeared more harmful than existing packs due to the large, clearly printed HWL. Most FGs found that the standard tin cylinder was less harmful than the standard paper sachet due to its comparatively smaller HWL.

**CONCLUSIONS:**

Both standard khaini packs increased HWL noticeability and perceived harm compared to existing packs, and the standard paper sachet was seen as more harmful than the standard tin cylinder. Implementing the standard sachet pack could enhance HWL visibility and increase perceptions of harm among consumers.

## INTRODUCTION

Smokeless tobacco (SLT) refers to a variety of tobacco-containing products consumed orally. Of the 300 million people who use SLT worldwide, approximately 200 million reside in India^1^. India also accounts for the bulk (70%) of the world’s SLT-related disease burden^2^, including oral cancers, reproductive health complications, stroke, and cardiovascular diseases^3^. SLT products are widely accepted in Indian culture and commonly offered at social occasions^2^. More than one in five adults in India uses SLT and 11% of adults consume khaini, a tobacco-lime SLT mixture^1^. Rates of khaini use are highest among men, lower socio-economic status populations, and rural populations^1^. Most Indian people who use SLT initiate use between the ages of 18 and 34 years^1^.

In 2004, India ratified the WHO Framework Convention on Tobacco Control (FCTC) and has since adopted policies to address SLT use. The Cigarettes and Other Tobacco Products Act (COTPA), a tobacco control law that prohibits tobacco advertising and regulates the trade, production, supply, and distribution of tobacco products, was enacted by the Government of India in 2003. COTPA mandated that SLT and all tobacco products sold in India display health warning labels (HWLs) that cover 85% of the front and back panels of the pack^4^. It also requires that HWLs be printed legibly, prominently, and in colors that contrast ‘conspicuously with the background’ of the label^4,5^.

When correctly implemented, HWLs have the potential to increase harm perceptions and reduce product use. In India, 46.2% of people who currently use SLT reported thinking about quitting smoking because of a HWL^1^. Another study conducted among people who smoked in Mangalore, India, found that 71.5% of survey respondents had thought about quitting as a direct result of HWLs^6^. Additionally, respondents who noticed pictures about the dangers of smoking were more likely to be knowledgeable about the health consequences of smoking, and they were more likely to consider quitting versus those who did not notice HWLs^6^.

Despite the role that HWLs can play in discouraging tobacco consumption, research suggests there are low rates of compliance with HWL placement on smokeless tobacco products in India^7,8^. Issues related to packaging and HWL compliance extend across multiple SLT products, including khaini. A 2017 assessment of SLT packs sold in 5 Indian states found that only about one-third of SLT packs (36%) were compliant with HWL location requirements and 98% did not meet that 85% HWL size coverage^9^. Most SLT packs also had a HWL that was distorted and/or printed in the wrong colors as required by law^9^.

One possible factor contributing to low HWL compliance may be the wide variety in the pack material (plastic, paper, tin), shape (sachet, cylinder), and size of SLT products sold in India^9^. Implementing a standard pack size and shape could improve correct application of HWLs on tobacco products. Previous research has shown that SLT packaging can be visually appealing and branded in ways that enhance product attractiveness and reduce perceived risk^10^. Standard packaging could reduce the space for attractive industry branding, which is known to increase the attractiveness of tobacco products for current and non-users and increase product initiation and sustained use^11-14^. To date, limited research is available regarding how standard SLT packaging may influence product appeal and harm perceptions in the context of India, where SLT use is highest and compliance with HWL requirements is low. We aimed to address this gap by qualitatively exploring perceptions of current khaini packs and two options for standard shape khaini packs among people who do and do not use SLT.

## METHODS

In March 2023, a total of 24 focus group (FG) discussions with 157 participants were conducted across three Indian states: Jharkhand, Maharashtra, and Utter Pradesh (Table 1). These states were chosen to reflect diverse areas of India; they are also states with rates of SLT use and khaini use that are higher than the national average^15^. Each FG lasted approximately 1.5 hours and included six to seven participants. Participants were recruited from low socioeconomic-status areas, given high rates of use among lower income populations^1^. FGs were numbered equally across urban/rural residence and gender to ensure even representation.

**Table 1:** Distribution of focus groups and participants by region, urbanicity, gender and tobacco use status

	*Jharkhand*		*Maharashtra*		*Uttar Pradesh*		*Total*
	*Urban*	*Rural*	*Urban*	*Rural*	*Urban*	*Rural*	
**Current khaini users**							
**Male**							
Groups	1	1	1	1	1	1	6
Participants	7	6	7	7	6	6	39
**Female**							
Groups	1	1	1	1	1	1	6
Participants	7	7	7	6	6	5	38
**Never tobacco users**							
**Male**							
Groups	1	1	1	1	1	1	6
Participants	7	7	7	7	6	6	40
**Female**							
Groups	1	1	1	1	1	1	6
Participants	7	7	7	7	6	6	40
**Total**							
Groups	4	4	4	4	4	4	**24**
Participants	28	27	28	27	24	23	**157**

This research was approved by the Johns Hopkins Bloomberg School of Public Health Institutional Review Board (IRB No 22331) and The Union Ethics Advisory Group. Participants provided informed oral consent and received a US$10 gift card as compensation for their participation.

### Sampling approach and recruitment

Using a systematic sampling approach (Supplementary file Table 1), participants were recruited from predetermined neighborhoods across the three states. For each selected neighborhood, a recruiter identified a random starting point (e.g. local market/store, central landmark, prominent crossroad) and then visited every other household. Recruiters enrolled only one eligible individual per household and used a pre-programmed survey on their smart devices to determine eligibility. Eligibility criteria included identifying as male or female, currently residing in the identified neighborhood, and proficiency in local regional language, including Hindi or Marathi. For FGs involving people who use khaini, participants had to be aged ≥18 years and report use of khaini in the past 30 days. For FGs with people who never used tobacco, participants had to be aged 18–34 years and report never using any tobacco product. We restricted the age of those who never used tobacco products to <35 years to capture those most at risk of tobacco initiation^1^.

### Data collection

A team of five trained moderators facilitated the FGs, following a structured discussion guide which included pack rating activities (Table 2).

**Table 2:** Focus group discussion guides on perceptions of current and proposed standard smokeless tobacco (SLT) packaging in India

Focus group	Discussion guide
**Noticeability of health warning labels on current khaini packs**	Now we are going to look at all products along with the scale for how much you notice the health warning labels on the product packaging. On one end of the scale is ‘most noticeable’ and on the other end of the scale is ‘least noticeable’. Look over the product packs again. Then write down your thoughts on where on the noticeability scale these product packs fall or place the packs directly on your paper scale. Again, we will discuss as a group. I have my own version of the noticeability scale where I will place the packs where most of the group thinks they belong. **Participants view khaini products and make notes/place on scale** How would you group these packs in terms of how much you notice the health warning label? Why do the warning labels on these packs stand out more? What makes them more noticeable? o *Probe*: Warning label placement on pack? Color? Image type? Image quality (sharp, blurry, etc.)? Why do the warning labels on these packs stand out the least? What makes them less noticeable? o *Probe*: Warning label placement on pack? Color? Image type? Image quality (sharp, blurry)? Would anyone rank these packs differently? Why?
**Perceived harmfulness of current khaini packs**	**Ask participants to remove noticeability scale and replace it with the harmfulness scale** Now we are going to look at all products together along with the scale for product harmfulness. On one end of the scale is ‘most harmful’ and on the other end of the scale is ‘least harmful’. Look over the product packs and consider the whole pack including the warning labels. Then write down your thoughts on where on the harmfulness scale these product packs fall or place the packs directly on your paper. We will discuss as a group, and I will place the packs where most of the group think they belong on my own version of the harmfulness scale. **Participants view khaini products and make notes/place on scale** How would you group these packs in terms of harmfulness? Why are these packs grouped as the most harmful? And these the least harmful? (e.g. descriptors, color/shine, images, flavor, product type etc.) Does anyone disagree with this grouping? What packs would you move around? How does the type of health warning label on the pack influence your grouping? How does the placement or visibility of the health warning label on the packs influence your grouping?
**Noticeability of health warning labels and harm perceptions of proposed standard khaini packs**	**Ask participants to set aside their sets of current khaini packs and open their set of the proposed standard product pack options for khaini** Next, we are going to discuss the product packaging option to standardize the shape, size, and material of khaini products. Please spend a few minutes looking at these products and their proposed price. Then we will ask you some questions about these packs. **Hold up Product A (standard khaini A: rectangular packet made of paper) and Product B (standard khaini B: cylinder shape, tin)** Compared to the existing products, how noticeable is the warning label on Product A? Why? Now Product B? Why? o *Probe*: Warning label placement on pack? Color? Image type? Image quality (sharp, blurry, etc.)? Compare Product A to Product B. On which pack is the warning label more noticeable? Why? How harmful is Product A compared to the existing khaini products we looked at? Why? How harmful is Product B compared to the existing khaini products? Why? o *Probe*: What features make Product A or Product B seem more or less harmful? o *Follow-up* (if not discussed): To what extent, does the placement of the warning labels on Product A and Product B influence how harmful these products seem? Compare Product A to Product B. Which product seems more harmful?

**Figure 1:**
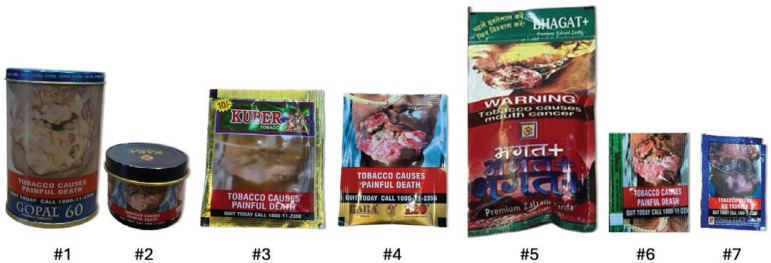
Images of 7 current khaini packs provided to focus group participants for pack rating activity and discussion (scaled to relative size)

**Figure 2:**
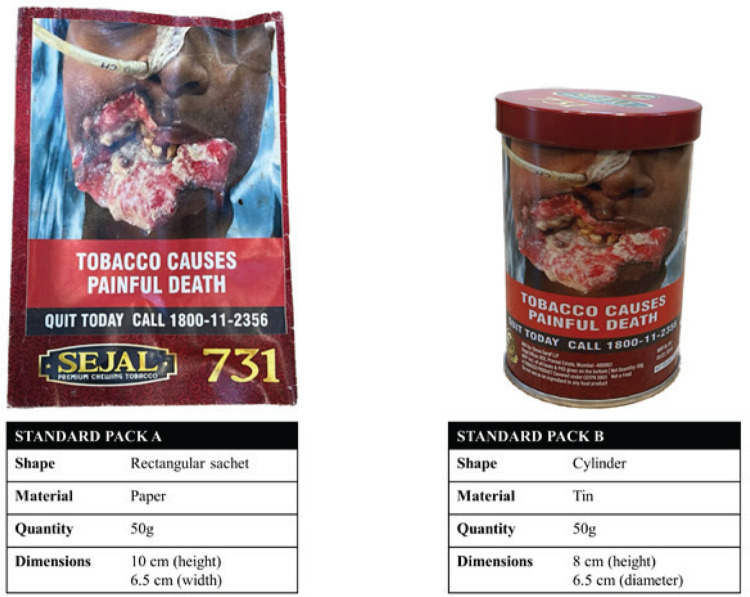
Images of the two standard packs with design specifications

Participants were provided with individual sets of the most popular Khaini products (n=7) available across India, or ‘current packs’ (Figure 1), and the two standard khaini packs: a paper sachet and a tin cylinder (Figure 2). The standard pack dimensions and material were developed in consultation with local tobacco control experts and both standard packs included the current HWL image rotation for tobacco products, which was placed on the top 85% of the front and back of the principal display area. The branding of the standard packs was similar and replicated what participants might typically come across in the market; however, the brand itself was fictional and not available for purchase. All packs were labeled, and participants referred to each pack by number or letter during the discussion.

Participants were given two scales to facilitate focus group discussion by rating the HWL noticeability and the perceived harm of the khaini packs. First, participants were asked to rate the current packs on a scale from 1 to 5 for HWL noticeability (1 = not very noticeable, to 5 = very noticeable). Afterward, the group rated the packs together on the same scale and then discussed their reasons for the group rating. This process was repeated for the perceived harm scale (1 = least harmful, to 5 = most harmful). FG ratings for the current packs were broadly consistent with the qualitative themes that emerged. After discussing the current khaini packs, each FG discussed how: 1) standard packs compared with current packs, and 2) compared the standard packs to each other in terms of HWL noticeability and perceived harm. Following the discussion, participants completed a brief questionnaire, including demographic and tobacco use information (Table 3).

**Table 3:** Participant demographics, past year quit attempts, and intention to use smokeless tobacco (SLT) among participants, by tobacco use status (N=157)

	*Current khaini users (N=77)*	*Never tobacco users (N=80)*
	*Mean (range)*	*Mean (range)*
**Age** (years)^a^	38.9 (20–70)	24.5 (18–34)
Male	39.6 (20–70)	23.5 (18–33)
Female	38.2 (22–60)	25.5 (18–34)
	***n (%)***	***n (%)***
**Education level**		
Less than primary school	10 (13.0)	5 (6.3)
Primary but less than secondary school	47 (61.0)	22 (27.5)
Secondary and higher	20 (26.0)	47 (58.8)
No formal schooling	-	6 (7.5)
**Occupation** ^b^		
Government employee	2 (2.6)	4 (5.0)
Non-government employee	11 (14.3)	12 (15)
Self-employed	37 (48.0)	17 (21.3)
Student	1 (1.3)	19 (23.8)
Homemaker	21 (27.3)	25 (31.3)
Retired/unemployed	2 (2.6)	3 (3.8)
**Past year quit attempt**		
Yes	22 (28.6)	-
No	55 (71.4)	-
**Intention to try SLT**		
I already use smokeless tobacco	64 (83.1)	-
Definitely yes	8 (10.4)	-
Probably yes	2 (2.6)	-
Probably not	-	-
Definitely not	1 (1.3)	80 (100)
Don’t know	2 (2.6)	-

a Age not given for 6 participants.

b Missing occupation for 3 participants.

### Coding and data analysis

All FGs were audio-recorded with participant consent, and a professional service transcribed the audio recordings into Hindi or Marathi, followed by translation into English. A subset of original and translated FG transcripts was reviewed by a researcher familiar with local languages and public health terminology for accuracy and consistency.

Based on the cursory review of the transcripts and internal discussions, a penultimate draft of the codebook was developed for test coding. Three authors (SS, RZ, HEB) coded the same three randomly selected transcripts to assess intercoder reliability. Any discrepancies during the test coding were discussed by the coders and author LC, and the codebook was finalized. Coders applied the final codebook to all 24 transcripts using the qualitative analysis software MAXQDA 2022. We conducted a thematic analysis of the coded data across dimensions of HWL noticeability and perceived harm using a guided approach to thematic analysis as outlined by Braun and Clarke (2006)^16^ (see final codebook in Table 4). We present results via summaries and exemplary quotations, which were edited for clarity.

**Table 4:** Final codebook applied to all 24 transcripts

Code name	Description	How to use
**Health warning label (HWL) Noticeability**		
**HWL size**	Discussion of how the size of the HLW text, HWL image, or the whole HWL on the pack enhances or limits one’s ability to notice the HWL.	Apply only to discussion of the HWL size in relation to the visibility or noticeability of the HWL. Can apply to both direct and implied comments and include words/phrases indicating positive or negative impact of the HWL size on HWL visibility.
**HWL placement**	Discussion of how the placement (e.g. top, bottom, around, broken) of the HWL on the pack enhances or limits one’s ability to notice the HWL. This includes how the shape of the pack influences where and how a HWL can be placed on the pack.	Apply only to discussion of the HWL placement in relation to the visibility or noticeability of the HWL. Can apply to both direct and implied comments and include words/phrases indicating positive or negative impact of the HWL placement on HWL visibility.
**HWL color**	Discussion of how the color of the HWL alone and in combination with the other colors on the pack enhances or limits one’s ability to notice the HWL.	Apply only to discussion of the HWL color or combination of the HWL color/other pack colors in relation to the visibility or noticeability of the HWL. Can apply to both direct and implied comments and include words/phrases indicating positive or negative impact of color on HWL visibility.
**HWL image quality**	Discussion of how the print quality of the HWL graphic (e.g. sharp, blurry, stretched, faded, tinted) enhances or limits one’s ability to notice the HWL. Image quality includes both HWL text, color (e.g. dark, bright printing), and pictorial image.	Apply only to discussion of the HWL image quality in relation to the visibility or noticeability of the HWL. Can apply to both direct and implied comments and include words/phrases indicating positive or negative impact of HWL image quality on HWL visibility.
**Language literacy**	Discussion of the ability to comprehend the image or text of the HWL enhances or limits one ability to notice the HWL. Literacy relates to not being able to read at all or an ability/inability to read a particular language (e.g. English).	Apply only to discussion of ability to read or comprehend (in the case of inability to read) the HWL message (text plus pictures). Can apply to both direct and implied comments.
**Perceived harmfulness**		
**Descriptors**	Discussion of how any of the textual elements (e.g. brand name, low tar) included on the pack increase or decrease perceived harmfulness of the product.	Apply only to discussion of the text included on the product pack and its relationship with perceived harmfulness. Can apply to both direct and implied comments and include phrases indicating positive or negative impact of text on harmfulness. Do not apply to HLW text; use ‘Harmfulness – HWL text’.
**Quantity**	Discussion of how the quantity of the tobacco product or size of the pack increase or decrease perceived harmfulness of the product.	Apply only to discussion of tobacco product quantity in relation to perceived harmfulness. Can apply to both direct and implied comments and include words/phrases indicating positive or negative impact of product quantity on harmfulness.
**Color**	Discussion of how the colors used on the pack increase or decrease perceived harmfulness of the product.	Apply only to discussion of the colors used on the product pack and its relationship with perceived harmfulness. Can apply to both direct and implied comments and include words/phrases indicating positive or negative impact of color on harmfulness. Do not apply to HWL colors; use ‘Harmfulness – HWL image’.
**Imagery**	Discussion of how the images included on the pack increase or decrease the perceived harmfulness of the product.	Apply only to discussion of the images and their relationship to perceived harmfulness. Can apply to both direct and implied comments and include words/phrases indicating positive or negative impact of imagery on harmfulness. Do not apply to HWL imagery; use ‘Harmfulness – HWL image’.
**Product type**	Discussion of how the type of product (e.g. zordha, gul, other smokeless tobacco, etc.) influences harm perceptions.	Apply only to discussion of tobacco product type in relation to perceived harmfulness. Can apply to both direct and implied comments and include words/phrases indicating positive or negative impact of product type on harmfulness.
**Product material**	Discussion of how the material of the packaging (e.g. paper tin) influences harm perception.	Apply on to discussion of product material in relation to perceived harmfulness. Can apply to both direct and implied comments and include words/phrases indicating positive or negative impact of product type on harmfulness.
**Product flavor**	Discussion of how the flavor, taste, or aromatic smell of the product (e.g. herbal, fruit, etc.) influences harm perceptions.	Apply only to discussion of flavor, including taste and smell, in relation to perceived harmfulness. Can apply to both direct and implied comments and include words/phrases indicating positive or negative impact of product flavor on harmfulness.
**HWL size**	Discussion of how the size of the HLW text, HWL image, or the whole HWL on the pack influences harm perceptions.	Apply only to discussion of the HWL size in relation to perceived harmfulness. Can apply to both direct and implied comments and include words/phrases indicating positive or negative impact of the HWL size on harmfulness. May be used when coding sections on Attractiveness, Noticeability, Behavioral Intentions when HWL elements are specifically discussed in relation to harm perceptions only. Use other codes if participants discuss HWL elements in terms of product attractiveness or HWL noticeability.
**HWL image**	Discussion of how the HWL color and imagery increases or decreases harm perceptions, including a lack of HWL image.	Apply only to discussion of the HWL color/imagery in relation perceived harmfulness. Can apply to both direct and implied comments and include words/phrases indicating positive or negative impact of HWL image on harmfulness. May be used when coding sections on Attractiveness, Noticeability, Behavioral Intentions when HWL elements are specifically discussed in relation to harm perceptions only. Use other codes if participants discuss HWL elements in terms of product attractiveness or HWL noticeability. If it is unclear whether the participant is discussing the HWL image or HWL text (e.g. ‘it looks clear’), then code for both HWL image and HWL text.
**HWL text**	Discussion of how the HWL text increases or decreases harm perceptions, including a lack of HWL text.	Apply only to discussion of the HWL text in relation perceived harmfulness. Can apply to both direct and implied comments and include phrases indicating positive or negative impact of HWL text on harmfulness. May be used when coding sections on Attractiveness, Noticeability, Behavioral Intentions when HWL elements are specifically discussed in relation to harm perceptions only. Use other codes if participants discuss HWL elements in terms of product attractiveness or HWL noticeability. If it is unclear whether the participant is discussing the HWL image or HWL text (e.g. ‘it looks clear’), then code for both HWL image and HWL text.
**HWL placement**	Discussion of how the placement of the HWL (e.g. top, bottom, around, broken) on the pack increases or decreases harm perceptions.	Apply only to discussion of the HWL placement in relation perceived harmfulness. Can apply to both direct and implied comments and include words/phrases indicating positive or negative impact of HWL placement on harmfulness. May be used when coding sections on Attractiveness, Noticeability, Behavioral Intentions when HWL elements are specifically discussed in relation to harm perceptions only. Use other codes if participants discuss HWL elements in terms of product attractiveness or HWL noticeability.
**Administrative**		
**Standard only**	Administrative code that will flag any discussion about the standard SLT pack alone (i.e. without any comparison to existing SLT packs).	Should be double coded with one or more of the Attractiveness, Noticeability, Harmfulness, Behavioral Intention codes.
**Standard vs existing**	Administrative code that will flag any discussion where participants make direct comparisons between the standard pack and existing SLT pack.	Can apply to comparison made with any existing gul or zordha products. Comparison product(s) do not need to be the current packs participants reviewed in the study but can include any pack on the market (i.e. not in the study). Should be double coded with one or more of the Attractiveness, Noticeability, Harmfulness, Behavioral Intention codes.

## RESULTS

### Participant characteristics

A total of 157 individuals participated in 24 FGDs across three Indian states. Across FGs, there were 77 participants who were current khaini users and 80 participants who had never used tobacco. Male and female participants were equally represented within each group, and FGDs were conducted in both urban and rural neighborhoods in all three states (Table 1).

Participant demographics are presented in Table 3. Individuals who use khaini had a higher mean age (38.9 years) compared to those who have never used tobacco (24.5 years), with minimal gender differences within each group. Among khaini users, 28.6% reported attempting to quit in the past year. Most (83.1%) reported current use of smokeless tobacco, while all individuals who have never used tobacco indicated they had no intention to initiate use.

Education level varied with 74% of individuals who use khaini having not completed secondary education, whereas 59% of those who have never used tobacco having completed secondary education or higher. Nearly half (48%) of individuals who use khaini were self-employed, and over a quarter (27.3%) identified as homemakers. Among individuals who have never used tobacco, the most common occupations were student (23.8%) and homemaker (31.3%).

Results were consistent across FG by residence, gender, and SLT use. We use the term ‘most’ to indicate themes that emerged in ≥80% of the focus groups.

### HWL noticeability

*Current packs*

For the current packs on the market, most FGs discussed the limited visibility of HWLs, noting that images were often too small and the printed HWLs images on packs were blurry, faint/printed in light colors, or dull/printed in dark colors. A respondent described a negative impact to the visibility of the HWL due to the size and shape of the packet:

Respondent: *‘The warning sign is not clearly seen because the packet is small. You can’t notice it because of the shape of the packet ... The picture should be complete of the face which is not given.’* (Khaini user group, Women, Rural Maharashtra)

When images were unclear, it lessened the comprehension, impact, and noticeability of HWLs.

Some groups expressed that current packs with excessive or poorly applied color – such as oversaturation or very light tones – reduced the visibility of HWLs. Packs with dull, washed-out images or overly bright, unnatural hues were sometimes disliked and perceived as less effective at drawing attention to the warning. These color distortions detracted from both the aesthetic appeal and the communicative power of the warning labels.

*Current versus standard packs*

With respect to the two standard khaini packs, both the shape and size of the standardized mock packs enhanced the visibility of the HWL image and text. All FGs discussed how the HWLs on standard pack A (sachet) and standard pack B (cylinder) were more noticeable than those on the current packs. One participant highlighted the enhanced noticeability and clarity of the HWL on standard pack A in comparison to all current packs:

Moderator: *‘… compared to the products that are available in the market how [visible is] the warning on product A?’.* Respondent: *‘Because of the pictures product, A is more noticeable and more clear. The other packets have small pictures.’* (Khaini non-user group, Women, Rural Maharashtra)

Further, standard packs featuring red prominently were described as more noticeable and visually striking:

Moderator: *‘Which is more noticeable warning?’* . Respondent: *‘The picture is big and bright. All red color. The red color is not looking nice.’* (Khaini non-user group, Women, Rural Jharkhand)

Most groups noted that the standard pack HWL images and text were easier to see and easier to understand due to the larger size in proportion to branding. Groups also raised how the bold or contrasting colors of the standard pack warnings, particularly red, helped draw attention to HWLs.

*Standard pack A (sachet) versus standard pack B (cylinder)*

Almost all FGs discussed how the HWL on standard pack A was more noticeable than the HWL on standard pack B. Broadly, FGs described how standard pack A’s HWL appeared bigger, brighter, and clearer. A respondent explained the HWL was more noticeable on standard pack A compared to standard pack B due to image size and layout:

Moderator: *‘Which out of [product] A and B [do] you notice the warning more?’.* Respondent: *‘A’.* Moderator: *‘Why?’.* Respondent: *‘Because the photo is so large and spread out. It is a warning by itself that the mouth will become like this. Both are same but this photo is large’.* (Khaini user group, Women, Rural Jharkhand)

The differences in size and shape of the two standard packs seemed to enhance noticeability, such that the HWL on the rectangular sachet was more noticeable across groups compared to the cylinder can.

### Perceived harmfulness

*Current packs*

All FGs discussed how the size and placement of the HWL image, along with the image content, influenced perceived harm of the current packs. Current packs that had larger, clearly printed HWLs were viewed as more harmful compared to current packs with smaller HWLs and HWL images printed unclearly (regardless of size). Several FGs also noted how the shape of the pack and placement of the HWL on one side only of the current pack would lower harm perceptions, with one respondent noting the ability to hide the HWL due to the shape of a current pack:

Respondent: *‘If someone holds it [pack #] in this way then the warning will not be visible at all.’* Moderator: *‘It is because of the shape there is a chance of hiding the warning?’*. Respondent: *‘Yes’.* (Khaini non-user group, Men, Urban Uttar Pradesh)

Many FGs discussed how having an emotional or fearful response to HWL image impacted the perceived harmfulness (and potentially use intentions) of the current packs, particularly if the HWL was large and clearly printed. A group explained the impact of the HWL image on their perceptions of harm and intent to use the product:

Moderator: *‘What is it in this one that look so harmful?’*. Respondent A: *‘By its appearance it is so dangerous as it seen the picture the condition of his mouth is very horrible.’.* Respondent B: *‘No one will eat this.’* (Khaini user group, Men, Rural Jharkhand)

Several groups also discussed how the size of a current pack or quality of the pack material influenced harm perceptions. A few FGs felt that smaller packs were less harmful because there was less product to consume. In addition, some groups discussed that what was perceived as ‘cheap’ pack material (e.g. paper pack without a plastic coating) increased perceived harm because it was an indication of a poor product quality or inability to withstand weathering.

A combination of factors influenced perceived harm of currents packs, including HWL size, placement, and content, where smaller or poorly placed HWLs lowered perceived harm and the ability to noticed and understand the graphic image of a HWL increased harm. Other factors, such as size and quality of packaging, also influenced harm perceptions where smaller packs seemed less harmful because the HWL was not as noticeable and the small amount of product to consume lowered perceived risk of use.

*Current versus standard packs*

With respect to the two standard khaini packs, a few groups noted that the size of the HWL image on the standard packs was larger compared to the branding, which increased the sense of danger and consequences of use among respondents:

Moderator: *‘In both these packs what is different than the market products that you feel it is most harmful?’.* Respondent A: *‘The picture that is given here is very dangerous.’*. Respondent B: *‘They have zoomed in and given the picture.’.* Respondent C: *‘Compared to market product, the picture is very dangerous and painful.’* (Khaini non-user group, Men, Urban Jharkhand)

All FGs discussed how the standard packs appeared to be more harmful than the current packs due to the prominent size, placement, and clarity of the HWL images.

*Standard pack A (sachet) versus standard pack B (cylinder)*

All FGs discussed how the HWL present on standard pack A made the product appear more harmful than standard pack B. Most FGs highlighted how the size and prominent placement of the HWL on standard pack A enhanced the details of the mouth ulceration and subsequently made the danger of use more prominent. Of note, some FGs discussed how the paper used to construct standard pack A made it appear more harmful than standard pack B because the paper seemed lower quality and cheap. In contrast, these FGs described how the tin material of standard pack B seemed more durable and therefore able to keep the product inside safe.

## DISCUSSION

Results from this study provide insight into the factors influencing consumer perceptions of SLT packs sold in India. Overall, we observed that design elements, as well as pack material and shape, influenced HWL noticeability and perceived harm of product use. We found the print quality (color saturation and contrast), placement, and content of HWLs largely influenced harm perceptions across groups. Packs currently on the market with large HWL images were rated as having more noticeable HWLs across groups in this study. Most found these larger HWLs easier to understand, reinforcing the potential dangers of khaini use. In contrast, packs with small or illegible HWLs were less noticeable, and groups discussed diminished comprehension of SLT-related health harms due to these smaller HWLs. Small HWLs were often present on smaller packs, suggesting how implementing a larger standard SLT pack size can increase the area for proper HWL placement.

Our findings are consistent with global studies emphasizing the role of color^17,18^, as well as shape and branding of SLT, on influencing group perceptions on products by people who use or do not use SLT alike^14,19-21^. Our results support that HWLs are more effective when printed clearly, with high-resolution and saturated colors (e.g. red). Blurred, faded, or distorted HWL images – common on current packs – were cited as barriers to HWL visibility. Findings provide a foundation for print quality standards and highlight the need for consistent HWL placement, in conjunction with pack size and shape, to maximize the impact and effectiveness of HWLs on SLT packaging.

Comparatively, the two standard packs assessed in this study consistently outperformed the current packs in terms of HWL noticeability and perceived harm of use. Groups found that the HWL on the standard sachet pack (pack A) was more noticeable than the HWL on the standard cylindrical pack and largely attributed the sachet’s packs rectangular shape as the reason the HWL appeared larger and more prominently placed. These findings align with prior research suggesting that large graphic warnings on tobacco product packaging have a direct negative effect on consumers, including increased quit intentions and decreased use behaviors^22-25^.

Collectively, our findings suggest that a rectangular SLT pack shape may have the greatest impact on perceived harm and HWL noticeability for both those who do or do not consume SLT than packs currently on the market^10^. Further, current SLT packs sold in India are often packaged in paper, making the transition to a standard paper sachet a plausible policy approach. In addition, the material of the standard sachet pack was also viewed as more harmful compared to the tin material of the standard cylindrical pack because paper was seen as less durable. This further confirms that a paper sachet shape may be preferred to optimize HWL noticeability and perceived harm, while reducing any potential for a standard tin cylindrical design to be appealing to consumers.

### Limitations

This study is subject to several limitations. The recruitment methodology focused exclusively on individuals within the three participating Indian states, and we did not include youth and people aged >34 years who do not use tobacco in this study. It is possible that our findings do not reflect all never tobacco users or people who use SLT in other states. By design, we also only examined one SLT product, khaini. Further, we only tested two possible standard packaging options. It is possible that results would differ among a range of SLT product types and/or standard pack designs. FG transcripts utilized for coding and analysis were translated into English, introducing the possibility of nuance or context loss from the original language of discussion. Our results also pertain to hypothetical commentary on SLT packaging rather than real-world, action-based responses. Despite these limitations, we adopted a rigorous recruitment and analysis approach, ensuring rich findings with data saturation across a socioeconomically and geographically diverse sample. While our findings offer insight into user perceptions of standardized packaging for smokeless tobacco, this study did not examine regulatory frameworks, compliance mechanisms, or implementation strategies. These are important areas for research, particularly with respect to the feasibility and enforcement of standardized packaging policies in diverse regulatory environments^26^.

## CONCLUSIONS

The prevalence of SLT use in India poses a significant public health concern. Despite legislation in place requiring HWL placement on 85% of the principal area of SLT packs, compliance remains an issue, undermining the effectiveness of HWLs^9^. Results from this study support the argument for implementing a standard rectangular sachet SLT pack in India. Evidence from around the globe suggests that implementing a standard SLT pack size and shape offers a viable strategy to address low compliance with existing HWL regulations, diminish SLT product allure, and encourage cessation among current users by removing distinctive branding elements and emphasizing graphic warning label imagery^25,27,28^. Other measures such as plain packaging legislation, which would also require SLT packs to be sold in a single color (e.g. drab olive) and enhanced enforcement of HWL placement and printing at the point of manufacture should complement a standard pack requirement^28^. Our findings underscore the potential of a standard SLT pack size, shape, and material to enhance HWL noticeability and increase perceived harms of SLT products in India. Standard packaging has the potential to be a strategic intervention to reshape consumer perceptions and contribute to improved health outcomes.

## Supplementary Material



## Data Availability

The data supporting this research are available from the authors on reasonable request.
