# Supplementary figures and images for: Standardizing smokeless tobacco packs in India to enhance health warning visibility and harm perceptions
Source: Tob Induc Dis. 2025 Jun 30;23:10.18332/tid/205097. doi: 10.18332/tid/205097 (PMC12210237; doi:10.18332/tid/205097)

**Supplemental Table 1.** Systematic sampling approach used to recruit study participants

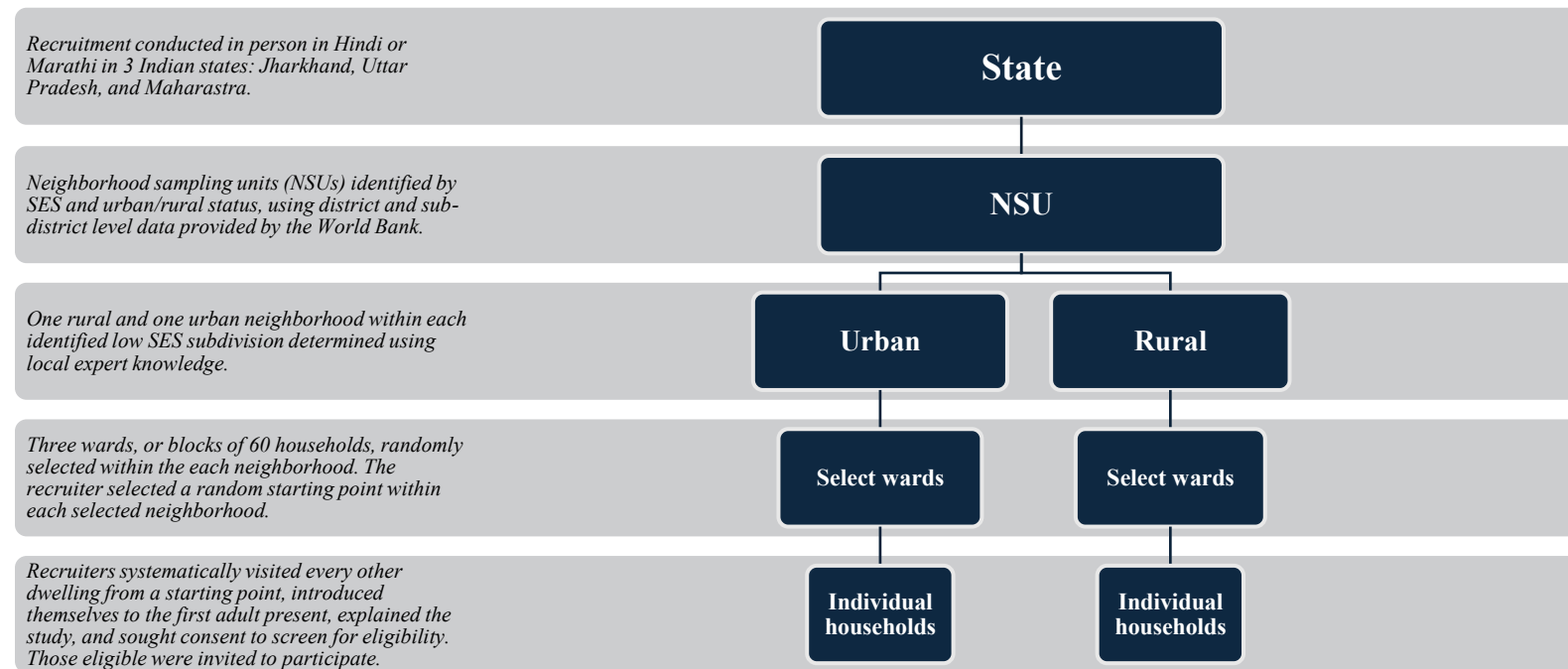

Supplement: Supplementary file 1 [file TID-23-87-s1.pdf]
